# Whole Plastome Sequences from Five Ginger Species Facilitate Marker Development and Define Limits to Barcode Methodology

**DOI:** 10.1371/journal.pone.0108581

**Published:** 2014-10-21

**Authors:** Justin N. Vaughn, Srinivasa R. Chaluvadi, Tushar, Latha Rangan, Jeffrey L. Bennetzen

**Affiliations:** 1 Department of Genetics, University of Georgia, Athens, Georgia, United States of America; 2 Department of Biotechnology, Indian Institute of Technology Guwahati, Assam, India; Chinese Academy of Medical Sciences, Peking Union Medical College, China

## Abstract

Plants from the Zingiberaceae family are a key source of spices and herbal medicines. Species identification within this group is critical in the search for known and possibly novel bioactive compounds. To facilitate precise characterization of this group, we have sequenced chloroplast genomes from species representing five major groups within Zingiberaceae. Generally, the structure of these genomes is similar to the basal angiosperm excepting an expansion of 3 kb associated with the inverted repeat A region. Portions of this expansion appear to be shared across the entire Zingiberales order, which includes gingers and bananas. We used whole plastome alignment information to develop DNA barcodes that would maximize the ability to differentiate species within the Zingiberaceae. Our computation pipeline identified regions of high variability that were flanked by highly conserved regions used for primer design. This approach yielded hitherto unexploited regions of variability. These theoretically optimal barcodes were tested on a range of species throughout the family and were found to amplify and differentiate genera and, in some cases, species. Still, though these barcodes were specifically optimized for the Zingiberaceae, our data support the emerging consensus that whole plastome sequences are needed for robust species identification and phylogenetics within this family.

## Introduction

The Zingiberaceae family includes ginger, turmeric, and cardamom spices, as well as many species with medicinal applications [1]. Although these plants have received little characterization at the genome level, there are many justifications for initiating such studies. Divergence in metabolite production in the Zingiberaceae can occur quite rapidly, exhibiting substantial variation even within the same species [2]. In contrast, morphological evolution is slower and often convergent [3]. Hence, DNA polymorphism analysis may be the most effective way to identify new germplasms that are sources of novel bioactive compounds. Previous work on the phylogenetic characterization of Zingiberaceae has focused on a small number of shared loci. While these loci can be used to differentiate genera, the approach fails to resolve genera into species [3]. Moreover, even for deeper phylogentic relationships, the Zingiberaceae contain many discrepancies between morphological and molecular classification of species; genera such as the *Alpinia* [4] and, to a lesser extent, *Curcuma* [3] are apparently paraphyletic.

Due to their high levels of conservation and maternal inheritance, chloroplasts can be used to develop population markers for phylogenetic and phylogeographic studies. The chloroplast DNA of most flowering plants are circular molecules that are highly conserved in terms of gene content, size and structural organization [5]. Because the size of the chloroplast genome is fairly constrained, highly conserved genes are found close to one another. These genes serve as optimal primer targets for amplifying short stretches of divergent intergenic DNA. In addition, chloroplasts outnumber nuclei in the plant cells. Thus, genes within the chloroplast are present in much greater concentrations than nuclear genes. While this may not be critical for analysis of fresh tissue, it can be a major consideration for dried specimens, gut samples, herbal stocks or other degraded DNA sources [6].

In addition to their pharmacological significance, gingers have become a model system for studying herbivore networks in tropical ecosystems through the use of DNA barcodes [7]. When the correct sequence region is chosen, DNA barcoding is an efficient way to assign an unknown specimen to a taxonomic group [6], [8]. In effect, finding that a specimen matches a previously characterized barcode allows a researcher to assign that specimen to the species associated with that barcode. Difficulties arise in taxonomic groups without variation across a chosen barcode. For example, Minami, *et al.* [9] used thirteen loci in an attempt to differentiate species within the paraphyletic *Curcuma* genus. Only one of the thirteen had haplotypes that were polymorphic across all accessions. Though these polymorphisms could be used to differentiate species, the intra-specific variation was similiar to the inter-specific variation, limiting the ability to assign unknown samples to higher taxonomic levels.

Both phylogenetic analysis and barcode classification are limited by the number of informative sites on which to base a conclusion. The entire chloroplast sequence dramatically increases the number of these sites, and, because of technological advances in sequencing, this increased information does not substantially increase total expenditures. Here, we present representative chloroplast genome sequences, or “plastomes”, for *Zingiber officinale* (ginger), *Amomum cardamomum* (cardamom), *Alpinia zerumbet* (shell ginger), *Hedychium coronarium* (white ginger), and *Curcuma longa* (turmeric). These data should serve as a foundation for further characterization of this pharmacologically significant lineage. Indeed, using these sequences, we developed a set of highly variable markers. We demonstrated the utility and limits of a subset of these novel markers on a range of Zingiberaceae species.

## Results and Discussion

### Assembly and annotation

**Figure 1:**
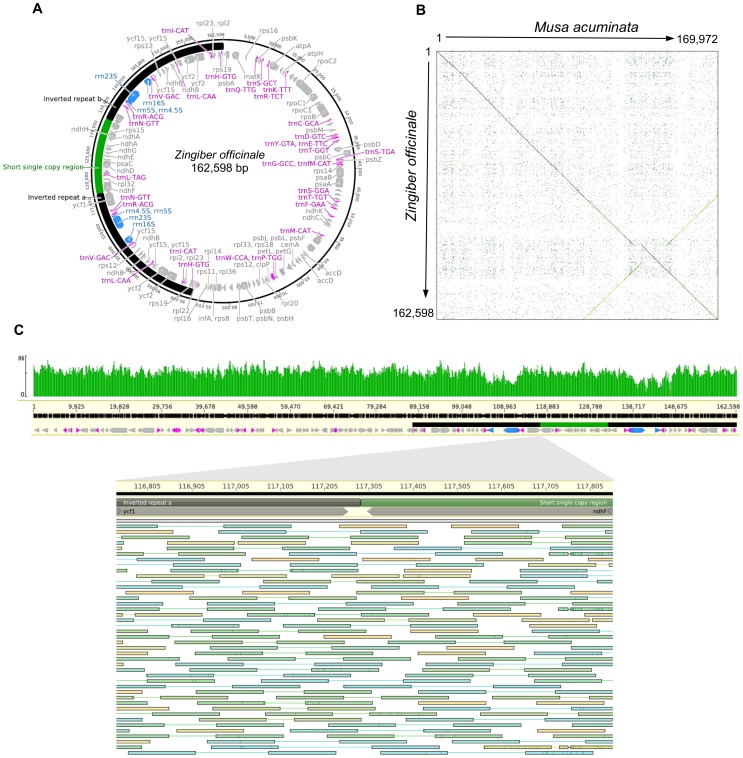
*Zingiber officinale* assembly quality and annotation. **A**, The Z. *officinale* plastome is shown in circular form with IRa and IRb indicated. The short single copy region between the two inverted repeats is shown in green. Protein coding genes are shown in grey, tRNA genes in pink, and rRNA genes in blue. *B*, Dotplot comparison of the *M. acuminata* and *Z. officinale* plastome sequences. The inverted repeat dotplot is transposed in gold. *C*, Total coverage across the *Z. officinale* plastome. The annotation color scheme is as depicted in A. The lower panel magnifies the border between the IRa and the SSC, where the large indel that differentiates *Musa* and *Zingiber* is seen to occur. Reads are shown individually; green is very near the expected insert size, while blue is slightly smaller and gold is slightly longer, respectively, than the expected size. The entire stack of reads is clipped due to space limitations.

We performed *de novo* assembly on the *Z. officinale* chloroplast genome using DNA sequences from a shotgun library of total cell DNA. Assembly resulted in two major contigs of 87,626 and 45,356. These were aligned to the *Typha latifolia* (gi|289065068|ref|NC_013823.1) plastome and scaffolded with smaller contigs. Borders between the inverted repeat regions (IRa and IRb) and the long and short single copy regions (LSC and SSC, respectively) were manually assembled and curated by iterative mapping of reads. A final plastome with 162,598 bp was produced (Figure 1A). Excepting a gap at the IRa/SSC border, the *de novo* assembly shows perfect colinearity with the *Musa acuminata* (banana) plastome [10] (Figure 1B). Sequencing reads are fairly even in their coverage of the entire plastome (Figure 1C) - as the IR regions are effectively identical, reads were randomly placed in either region.

Annotation was performed using CpGAVAS [11]. In general, there are no major discrepancies in the gene content of the *Z. officinale* plastome compared to known plastomes, excepting an expansion of the IRa region as described below. The expansion creates a duplication of the first 3,912 bp of the *ycf1* gene. YCF1 and YCF2 are the longest proteins encoded by known plastomes and appear to be indispensable to plant survival [12]. Though the *Zingiber* plastome encodes a full-length YCF1, it is unknown if the large fragment associated with the IRa/SSC boundary is also expressed.

### Repeat expansion and structural variation

The borders of inverted repeats and single copy regions are known to be hypermorphic for major structural variation. A major expansion of IRa has been noted in the *Musa* lineage [10]. As seen in Figure 1, the *Z. officinale* plastome does not completely share this expansion, although it does have a 3 kb expansion at this site relative to the otherwise highly colinear *Amborella trichopoda* (gi|34500893|ref|NC_005086.1) and *T. latifolia* plastomes (Figure S1 and S2). (Because of their deep ancestry, the colinear relationship between *Amborella* and *Typha*, among others, suggests that this is the ancestral plastome structure.) Thus, it appears that an initial 3 kb IRa expansion occurred in the *Musa*/*Zingiber* ancestor and was further expanded in the *Musa* lineage by another 4.7 kb. Alternatively, a 7.7 kb expansion could have occurred in the *Musa*/*Zingiber* ancestor followed by a deletion at the IRa/SSC boundary in the *Zingiber* lineage after divergence from the *Musa* lineage. The structure of the recently sequenced *Zingiber spectabile* plastome [13] suggests that these were separate events and that the short expansion has only occurred in *Z. officinale*. By mapping our sequencing reads from the *C. longa* plastome to the *Z. spectabile* scaffold, it is apparent that the scaffold has collapsed the 3 kb Zingiberale IRa expansion into the SSC. We see no such discrepancy in coverage when reads from *C. longa* or the more distantly related *A. cardamomum* are mapped to the *Z. officinale* plastome. Since this small expansion appears to have occurred at the root of the Zingiberaceae, the large expansion in *Musa* may have occurred in multiple steps. Whether this sequence is predisposed to expansion or such expansions are preferentially retained by natural selection remain open questions.

### pan-Zingiberaceae variation

We then used the *Z. officinale* plastome as a reference on which to assemble data from four other libraries of lower coverage or shorter read length (Table 1). The final sequence reported for each plastome is the consensus sequence of these mapped reads (see Materials and Methods).

**Table 1:** General features of the material used for sequencing and the sequencing method.

Species	Site of Collection	Sequencing Method	Insert Size (bp)	Total Reads (10^6^)	Chloroplast Reads (10^3^)	Coverage
*Zingiber officinale* (TARS18100)	USDA Tropical Agriculture Research Station, Puerto Rico	MiSeq-PE150	400–600	3.0	52.9	50×
*Curcuma longa* (ZSD07)	Assam, India	MiSeq-PE150	400–600	1.3	58.1	54×
*Alpinia zerumbet* (TARS1718)	USDA Tropical Agriculture Research Station, Puerto Rico	HiSeq- PE100	400–600	3.0	35.4	22×
*Amomum cardamomum* (ZSB03)	Assam, India	HiSeq- PE100	400–600	10.8	112.4	70×
*Hedychium coronarium* (UGA-Hyd)	UGA Greenhouse, Athens, GA	HiSeq- PE100	400–600	20.2	337.1	211×

Generally, the IRs, which encode ribosomal RNAs, are highly conserved relative to both the LSC and SSC regions. Within the LSC and SSC, highly variable regions correlate with regions that either encode tRNAs or short genes and have a low gene density. Interestingly, regions of the IR with similarly low gene density exhibit much higher levels of conservation. This may be related to regulatory constraint outside of the protein coding regions or to a homology-dependent DNA repair mechanism specific to the IR region [14], [15].

**Figure 2:**
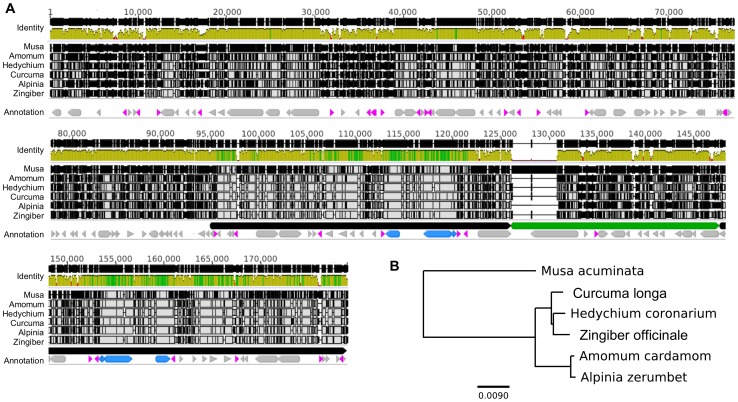
Plastome sequence variation across five Zingiberaceae. **A**, Alignment of whole plastome alignment between *M. acuminata* and Zingiberaceae species sequenced in this study. The annotation color scheme is as depicted in Figure 1A. In the coverage plot, color indicates percent identity: green, 100%; gold, >30%; red, <30%. Black bars within each species row indicate polymorphisms relative to the consensus. **B**, A phylogenetic tree based on the plastome alignments. *Musa acuminata* was used as the outgroup. Each branch point has 100% bootstrap support. Scale bar indicates the number of substitutions per site estimated to have occurred for such a length along the tree.

The tree generated from alignment of these plastomes (Figure 2) agrees with previous trees based on combined data from the nuclear ITS locus and the chloroplast *matK* gene [3]. Both trees indicate that *Hedychium* and *Zingiber* share a more recent common ancestor than *Zingiber* and *Curcuma*, although the divergence of all three lineages appears to have been nearly simultaneous on an evolutionary time scale.

### Microsatellite identification and barcode development

Short sequence repeats (SSRs) or “microsatellites” are hypermorphic due to errors during DNA replication. Such sequences often serve as useful loci for differentiating closely related individuals. In contrast, DNA barcoding exploits point mutations and short indels that occur in rapidly evolving sequences flanked by more slowly evolving sequences. The lower rate of mutation in these barcodes relative to SSRs is somewhat compensated for by the presence of multiple informative sites in a single barcode sequence. Which of these strategies is most appropriate depends on the application, the taxonomic group under study, and the available instrumentation. Indeed, a very short SSR may serve as one among many informative sites within a single barcode. Using *M. accuminata* plastome sequence, researchers were able to identify 15 chloroplastic SSR markers that were polymorphic within the species [10]. By using both the *Z. officinale* sequence and the additional Zingiberaceae data produced in this study and elsewhere [13], we attempted to generate reliable chloroplastic SSR markers using a comparative approach. Briefly, alignment information from a whole plastome comparison between *Z. officinale* and *Z. spectabile* was used to find di- and tri-nucleotide repeats that were polymorphic within the *Zingiber* genus. We avoided homopolymer repeats because, generally, they are technically difficult to use as markers. We also exploited alignment information in order to generate reliable primers for the amplification of these polymorphic regions. Using a similar pipeline for the alignment of all plastomes in this study (Figure 2A, excluding *Musa*), we also identified SSR markers that are likely to be most effective across the Zingiberaceae.

“Universal” plant barcodes, such as *matK* and *trnH-psdA*, have been tested across a range of Zingiberaceae. Shi *et al.* [16] found that the nuclear ITS2 region, because of its high interspecific divergence, was superior to universal choloroplastic barcodes. In contrast, Vinitha *et al.* [17] showed that the utility of both ITS and ITS2 regions is limited by low direct sequencing efficency caused by intragenomic heterogeneity. These researchers determined that *matK* remained the superior barcode for gingers, but *matK* failed to differentiate species in 25% of cases. Similiar findings in other taxonomic groups [18], [19] have led researchers to develop group-specific barcodes [20]. One aim of this study was to compare the discriminatory power of “universal” barcodes tested in the referenced works with barcodes that are designed specifically for gingers based on whole plastome sequences spanning the family.

To find suitable barcodes, we searched the alignments for regions that have at least 2% divergence. The regions were also filtered based on the number of single nucleotide polymorphisms (SNPs) that were bordered by 5 conserved bases on both sides of the SNP. Similar to SSR discovery, we identified markers using the intra-genus comparison for *Zingiber* as well as the comparison across all plastomes from this study. For the intra-genus and inter-genus comparison, we required at least 5 and 8 of such SNPs, respectively, across a 300 bp region. As with the SSR marker discovery, primers were only chosen from perfectly conserved sequence blocks.

**Figure 3:**
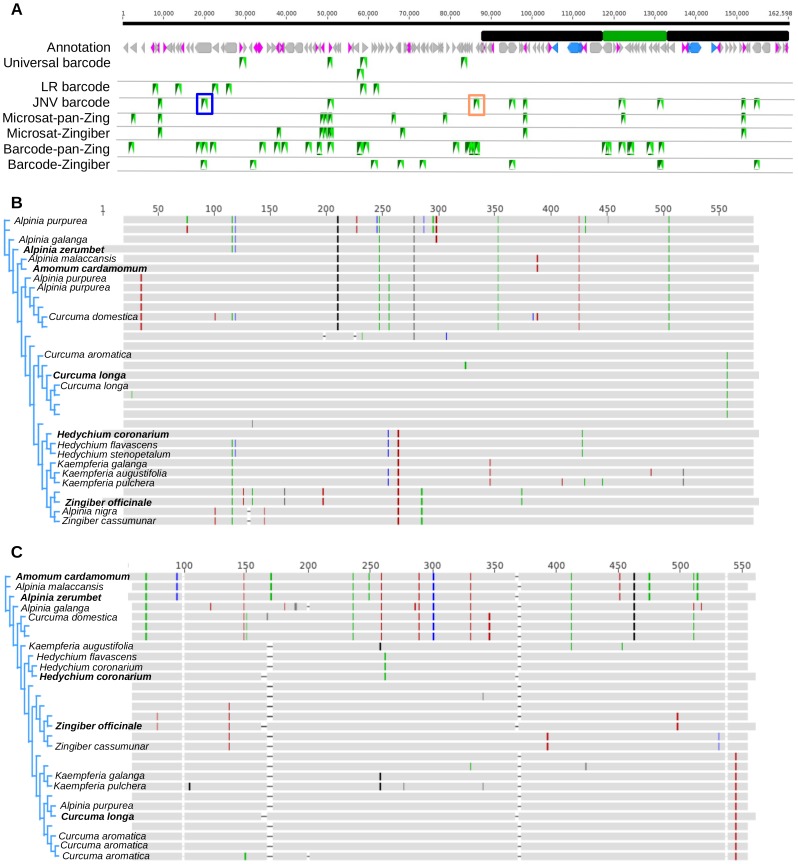
Assorted primer combinations from this and prior studies. **A**, “Universal barcode” primers are those described in [21]. “LR barcode” primers are from a recent unpublished study. “JNV barcode” primers were developed in this study and were generated based on the microsatellite and barcode primers identified by the computational pipeline described in the text. These are indicated in the figure as the last four tracts, where “Zingiber” indicates the intraspecific *Z. officinale* and *Z. spectabile* comparison and “pan-Zing” indicates a comparison across all sequences described in this study. Underlined markers indicate multiple overlapping markers at that site. The blue and orange boxes demarcate the markers used to generate sequences in Figure 3B and 3C, respectively. **B** and **C**, Sequences from a range of Zingiberaceae species were amplified using the primer pairs indicated in Figure 3A. **B** is associated with the blue box and shows products amplified by barcode set JNV-C (Table S1). **C** is associated with the orange box and shows products amplified by barcode set JNV-A (Table S1). Grey color indicates identity with the consensus of the alignment. Colored sites indicate a polymorphism, where different colors represent 4 different bases and light grey represents an ambiguous base. Appropriate regions from the fully sequenced plastomes were extracted and included in the alignment. These sequences are indicated by bold font. Names are not shown for samples that were not keyed to species-level.

The “Universal barcode” primers in Figure 3 are from previously proposed sets designed to work across all flowering plants [21]. Additionally, the “LR barcode” track plots another set of commonly used primer sets within the Zingiberaceae (Latha *et al.*, unpublished). Figure 3 also indicates the four sets of primer pairs resulting from our marker discovery pipeline. Looking across all four sets, we chose a subset of primers that exhibit a high number of polymorphisms at both the intra-genus and intra-family levels (“JNV barcode”). As shown, there is very little overlap between the “JNV barcode” markers and those used in previous studies. Though not shown in detail, only one of the “Universal barcode” primer sets perfectly matches the *Z. officinale* plastome, while the rest that are shown contain one to many mismatches. (Those with more than two mismatches are not shown.) While these mismatched primers may still produce a clear PCR product, they are not optimal with regard to heterogeneous starting material. Again, we aimed to make our primers as unbiased as possible by selecting from only highly conserved sites. Thus, in theory, “JNV barcodes” are optimal markers for the Zingiberaceae.

### Marker testing on multiple Zingiberaceae lineages

To demonstrate the utility of JNV barcodes (Table S1), we amplified and Sanger sequenced these regions from a range of previously characterized and uncharacterized Zingiberaceae specimens (Table S2). Generally, all pairs amplified across the Zingiberaceae phylogeny, but JNV-A and JNV-C exhibited superior performance under the conditions tested (Table S2, Figures S4, S5). These pairs were followed in efficacy by JNV-E, JNV-D, and JNV-B. Pairs JNV-G, JNV-H, and JNV-I failed to amplify in any of the tested samples. Importantly, all pairs were tested in a single experiment as a proof-of-concept; we expect that PCR optimization on a pair-specific basis would greatly improve their amplification efficiency and phylogenetic distribution. Pairs JNV-A and JNV-C were able to efficiently amplify sequences for all genera in this study (Table S2, Figures S4, S5). They resolve a major branch point between *Alpinia/Amomum* samples and *Zingiber/Hedychium/Curcuma* samples (Figure 3B–C). The latter group could generally be differentiated by one or more polymorphic sites into three subgroups coinciding with the three different genera. As predicted by the whole plastome alignment, the branch between the *Zingiber/Hedychium* and *Curcuma* split was very short and thus difficult to resolve (Figure 2B). Still, there are clearly polymorphisms in both data sets that cluster *Zingiber* and *Hedychium* to the exclusion of *Curcuma* (Figure 3B–C). In addition, these barcodes clearly discriminate species mis-identified as belonging to the *Alpinia* genus (Figure 3B–C).

In terms of species resolution, there are cases where two species can be resolved, such as within the *Hedychium* and *Zingiber* groups, as well as within the *Alpinia* group (Figure 3B–C and File S1 and S2). *Curcuma* species are more difficult to resolve using these primers. Either this is a result of broad similarity across the whole plastomes of this group, or the markers we have chosen do not correspond to the most variable loci within this group. Additional plastome sequences within the *Curcuma* should resolve this question. As observed in other studies [3], [4], the paraphyletic nature of genera such as *Alpinia* and *Curcuma* is also evident from our data, while genera such as *Zingiber*, *Kaempferia*, and *Hedychium* show monophyletic distributions (Figure 3B–C).

## Conclusions

There is growing consensus that the entire chloroplast genome sequence is an optimal ‘barcode’ in plant identification and phlyogenetics [22]–[25]. Our study supports this consensus: even markers optimized to focus on the most variable (but still alignable) regions of these plastomes failed to produce enough informative sites for differentiating the most closely related species and for generating a tree with comparable bootstrap support of the same nodes shown in Figure 2B. Moreover, given the low cost of next-generation sequencing, the price of sequencing an entire plastome versus amplifying and Sanger sequencing a single barcode are approaching equivalence. This is particularly true when a reference plastome is available to facilitate assembly using lower read coverage.

Still, even a comparison of entire plastome sequences will reach resolution limits as samples are drawn from very closely related individuals [23]. In terms of the *Zingiber officinale/spectabile* comparison, plastomes exhibit hundreds of genuine polymorphisms, while universal barcodes *matK* and *rbcL* possess only 4 and 2, respectively (Figure S3). Though the ginger-specific barcodes reported in this study, JNV-C and JNV-A, have at least 5 polymorphisms between these two species, they too are ineffective with regard to *Curcuma* species (Figure 3B–C), suggesting far fewer polymorphisms exist across plastomes of that genus. As discussed above, whole-plastome sequences from the *Curcuma* genus will be an interesting test case for the differentiating power of organellar sequences.

Short-read sequencing and assembly strategies will also be ineffective when faced with a heterogeneous pool of samples from similar species, as might be associated with an ecological study or with herbal/spice contamination detection. Both the former [7] and latter applications are of particular interest within the Zingiberaceae. Therefore, exploiting the range of chloroplast sequences presented in this study, we have also developed a new set of barcode markers optimized for the Zingiberaceae. Two of those tested primer pairs, individually resolve the major and minor branches within the family. Though closely related species could be resolved in some cases, a combination of these markers is still likely to be required for confident identification of the constituent species in a mixed pool.

## Methods

### Sampling of Zingiberaceae accessions

Zingiberaceae accessions used in the current study were collected from US and India (Table 1). They are being maintained in greenhouses at the University of Georgia, Athens, GA and the Indian Institute of Technology in Guwahati, Assam, India.

### DNA extraction and Illumina Sequencing

DNA extractions were carried out using PlantDNeasy mini kits (Qiagen, USA) following the manufacturer's instructions. The TruSeq sample preparation kit (Illumina, USA) was used to prepare DNA libraries with inserts from 400–600 bp for paired-end multiplexed sequencing. Library preparations and sequencing on an Illumina HiSeq2000 instrument were carried out at the Interdisciplinary Center for Biotechnology Research, University of Florida. Library preparations and sequencing on an Illumina MiSeq instrument were performed at the Georgia Genomics Facility, University of Georgia. Library-specific indexed adapter sequences were used to separate reads from individual samples from pooled libraries. All sequencing reads used in the assemblies have been made available in the Short Read Archive (http://www.ncbi.nlm.nih.gov/sra) under the BioProject accession PRJNA253694.

### Assembly and annotation

For the *Z. officinale* plastome, shotgun reads were assembled as follows. Total reads were first assembled using *edena* with default parameters. Reads where then filtered to remove read pairs with polynucleotide runs of >30 bp and consecutive Ns of >75. Contigs resulting from *edena* assembly were used as long pseudo-reads (‘-long’ parameter) in a second round of assembly by *velvet* in paired-end mode with *k*-mer size set to 51 bp and the minimum k-mer coverage set to 2 (‘-cov_cutoff 2’). Additionally, we required only 1 long read to scaffold two contigs (‘-long_mult_cutoff 1’). Masked reads were then mapped back to the final assemblies using *bowtie-2* in local mode (–local), and scaffolded gaps were manually curated. We arrived at this hybrid strategy - a combination of overlap-consensus-layout and *k*-mer graph approaches – by observing that, while *velvet* was more robust to sequencing errors and could extend contigs further, *edena* was less prone to make errors when resolving the ends of duplicated sequences. The CpGAVAS web service [11], based on the MAKER pipline [26], was used to annotate the *Z. officinale* plastome. The sequence with annotation has been deposited in GenBank (http://www.ncbi.nlm.nih.gov/genbank/) under the accession KM213122.

For the assembly of other species in this study, total shotgun reads from a given individual were mapped to the *Z. officinale* plastome using Geneious (6.0.6) with ‘Medium Sensitivity’ and 5 iterations. Matches with equal scores were randomly assigned to an appropriate position. A consensus sequence was generated such that 50% of the bases across all mapped reads were required to be identical. An ‘N’ was called if the quality score was <20. Total shotgun reads were remapped to these consensus sequences and manually curated. All sequence assemblies are available as supporting information (File S3).

The MAUVE aligner (‘mauveAligner’ algorithm) [27] was used to create whole plastome alignments between the *Musa acuminata* sequence (gi|525312436|embl|HF677508.1) and all Zingiberaceae sequences generated in this study. A seed weight of 8 was used, and plastomes were assumed to be colinear. Resultant alignments and PhyML were used to estimate the topology, branch length, and substitution rate of a tree relating these species. A Kimura (K80) substitution model was used with 50 bootstrap trials. *M. acuminata* was chosen as the outgroup.

### Identifying optimal markers

The method we employed to identify both microsatellite and barcode markers use an alignment to maximize both the variability of the marker and the consistency of the primer sites used to amplify the marker. To that end, two consensus sequences were generated from whole plastome alignments: the “best-base” consensus, for which the majority base in every column was used and gaps were ignored, and the “ambiguous” consensus, for which 100% of bases were required to match and gaps were considered. Thus, explicit bases (i.e., A, C, G, and T) in the ambiguous consensus are indicative of a perfectly conserved column.

For microsatellite marker development, we used the best-base consensus in conjunction with MISA software [28] to identify di- and tri-nucleotide repeats of 4 units or more. Using a custom Perl program, the resultant coordinates were then screened against the ambiguous consensus to determine if they were in fact polymorphic across the species analyzed, such that an ambiguous base was required to be present within the microsatellite or within 4 bp to the left or right of it. Primer3 [29] was then used to identify primer sites within the ambiguous consensus that amplify polymorphic positions, with the following parameters: PRIMER_PRODUCT_SIZE_RANGE = 100–280, PRIMER_MAX_END_STABILITY = 250, and PRIMER_LIBERAL_BASE = 1. Primers were not picked from ambiguous sequence, so, by using the ambiguous consensus, only highly conserved regions were considered.

A similar approach was used for barcode development, except that, instead of MISA software, a custom Perl program was written to find 300 bp blocks that 1) had >2% divergence, 2) did not contain more that 7 consecutive ambiguous bases, 3) did not have >10% or 15% divergence for genera or family comparisons, respectively, and 4) had >4 or >8 single-nucleotide polymorphisms with 5 conserved flanking bases for genera or family comparisons, respectively. Primer sequences were sought for within 300 bp upstream and 300 bp downstream of a 300 bp block passing the above criteria. The same parameters were used as for microsatellite primer identification (excepting the different size range). All programs not from external sources are available upon request.

### PCR analysis with novel barcode primers designed in the current study

Genomic DNA was isolated from 26 Zingiberaceae accessions collected from Assam, India and the USA. These accessions included 20 identified species and 6 samples whose species had not been determined. Table S2 lists details of the various accessions used in this study. We tested 11 primer pairs designed in the hypervariable regions of the chloroplast genome. All the primers used are listed in Table S1. Six primer pairs showed consistent amplification across most of the species. PCR products amplified with two primer pairs, A and C (Table S1), were used for Sanger sequencing. PCR reactions were performed in 50-µL reactions: 1× Phusion High Fidelity Buffer (Finzyme, Finland), (NEB, USA), 0.25 µM of each primer, 0.5 µM each dNTP, 1 U Phusion High-Fidelity DNA Polymerase (NEB, USA), and 100 ng DNA. PCR conditions were as follows: 98°C for 2 min; 26 cycles of 98°C for 10 s, 58°C for 20 s, 72°C for 30 s; with a final extension at 72°C for 10 min. Approximately 5 uL of the PCR reaction was tested on the gel to verify the presence of amplification product. PCR reactions were purified with the AGENCOURT AMpure kit (Beckman Coulter, USA) as per manufacturer's instructions. PCR product purification and direct sequencing were performed by standard procedures at the Georgia Genomics Facility (University of Georgia, Athens, GA) on an ABI 3730 instrument.

## Supporting Information

Figure S1 **Dot-plot alignment of** ***Zingiber officinale*** **and** ***Amborella trichopoda*** **plastomes.**
(PDF)

Figure S2 **Dot-plot alignment of** ***Zingiber officinale*** **and** ***Typha latifolia*** **plastomes.**
(PDF)

Figure S3 **Alignments for matK and rbcL barcode sequences from** ***Zingiber officinale*** **,** ***Zingiber spectabile*** **, and** ***Hedychium coronarium*** **.** Colored letters indicate polymorphic sites.
(PDF)

Figure S4 **Gel documenting amplification efficiency of JNV-C barcode sequence among Zingiberaceae accessions.** Lanes are coded based on the matrix given below the gel. Plant accessions are as they appear in Table S2.
(PDF)

Figure S5 **Gel documenting amplification efficiency of JNV-A barcode sequence among Zingiberaceae accessions.** Lanes are coded based on the matrix given below the gel. Plant accessions are as they appear in Table S2.
(PDF)

Table S1 **List of primers identified as optimal barcodes.** Shown as ‘JNV barcodes’ in Figure 3.
(DOC)

Table S2 **Species list and results for barcode A and C amplification.**
(DOC)

File S1 **Alignments of sequenced products from barcode A PCR in fasta format.**
(FASTA)

File S2 **Alignments of sequenced products from barcode C PCR in fasta format.**
(FASTA)

File S3 **Fasta file containing each assembled plastome sequence.**
(FAS)
