# Supplementary figures and images for: Whole Plastome Sequences from Five Ginger Species Facilitate Marker Development and Define Limits to Barcode Methodology
Source: PLoS One. 2014 Oct 21;9(10):e108581. doi: 10.1371/journal.pone.0108581 (PMC4204815; doi:10.1371/journal.pone.0108581)

# Amborella trichopoda

Zingiber officinale

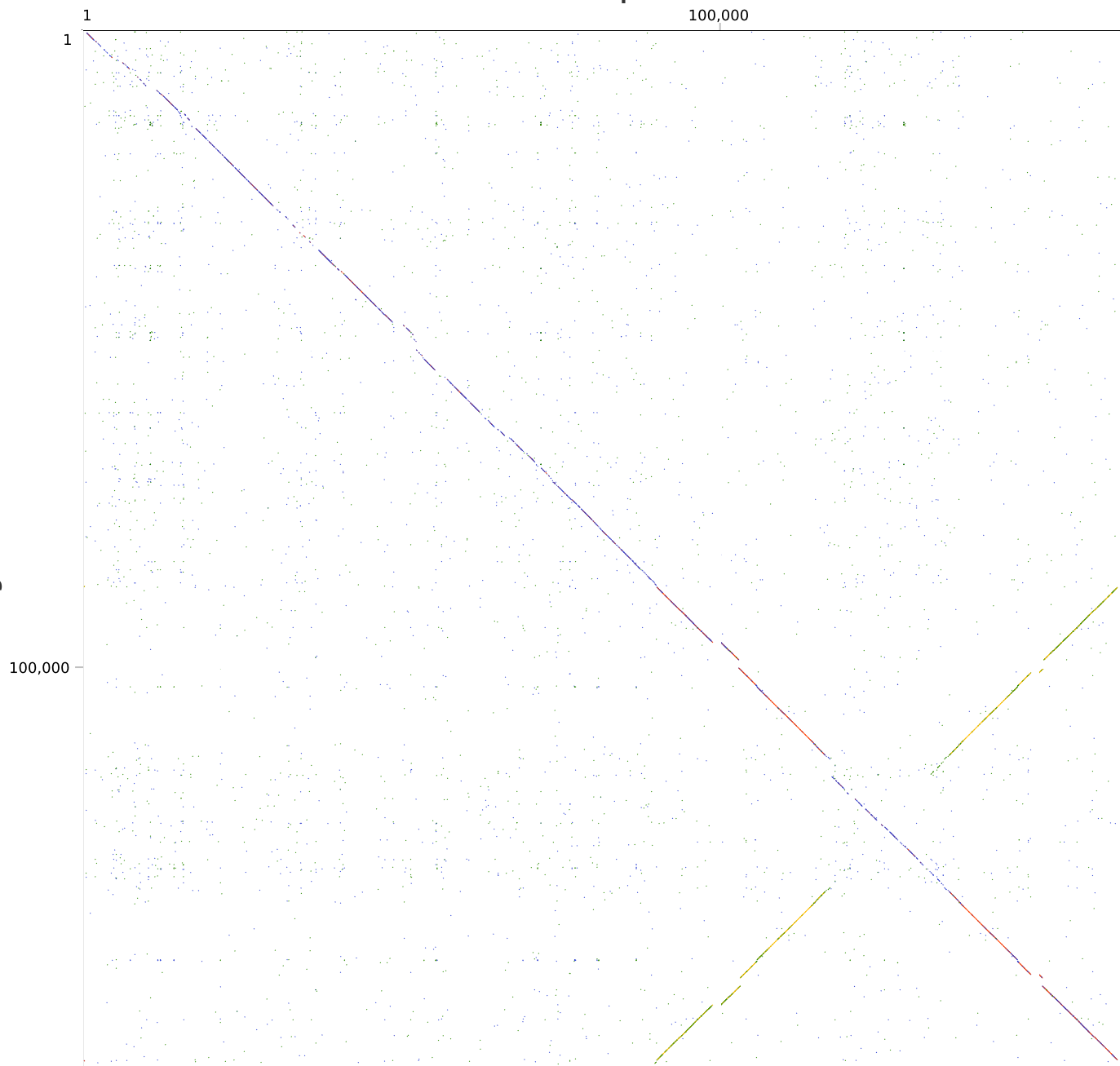

Supplement: Figure S1 — Dot-plot alignment of Zingiber officinale and Amborella trichopoda plastomes. (PDF) [file pone.0108581.s001.pdf]

# Zingiber officinale

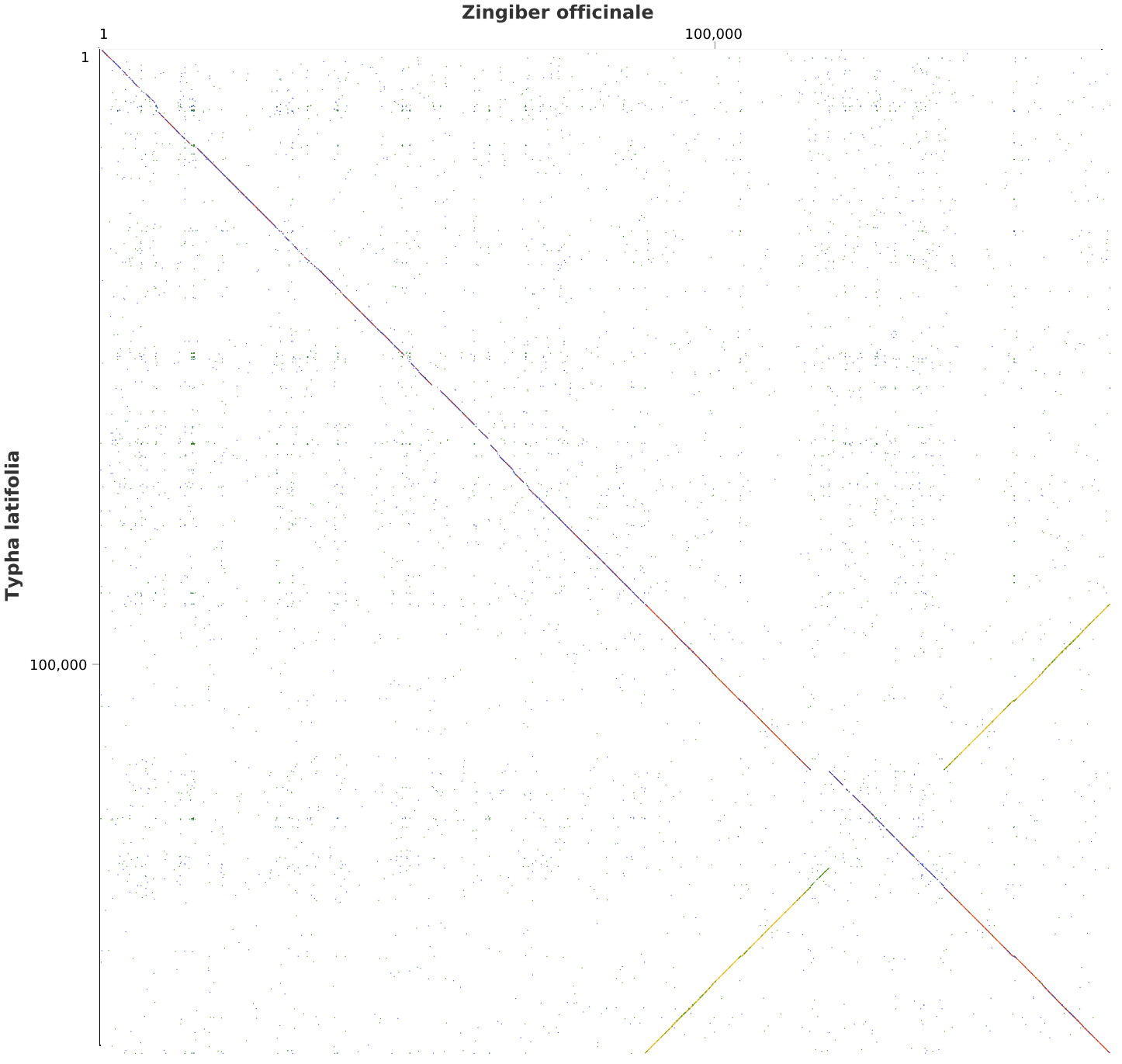

Supplement: Figure S2 — Dot-plot alignment of Zingiber officinale and Typha latifolia plastomes. (PDF) [file pone.0108581.s002.pdf]
