# Supplementary material for: Whole Plastome Sequences from Five Ginger Species Facilitate Marker Development and Define Limits to Barcode Methodology
Source: PLoS One. 2014 Oct 21;9(10):e108581. doi: 10.1371/journal.pone.0108581 (PMC4204815; doi:10.1371/journal.pone.0108581)
Supplement: Figure S3 — Alignments for matK and rbcL barcode sequences from Zingiber officinale , Zingiber spectabile , and Hedychium coronarium . Colored letters indicate polymorphic sites. (PDF) [file pone.0108581.s003.pdf]

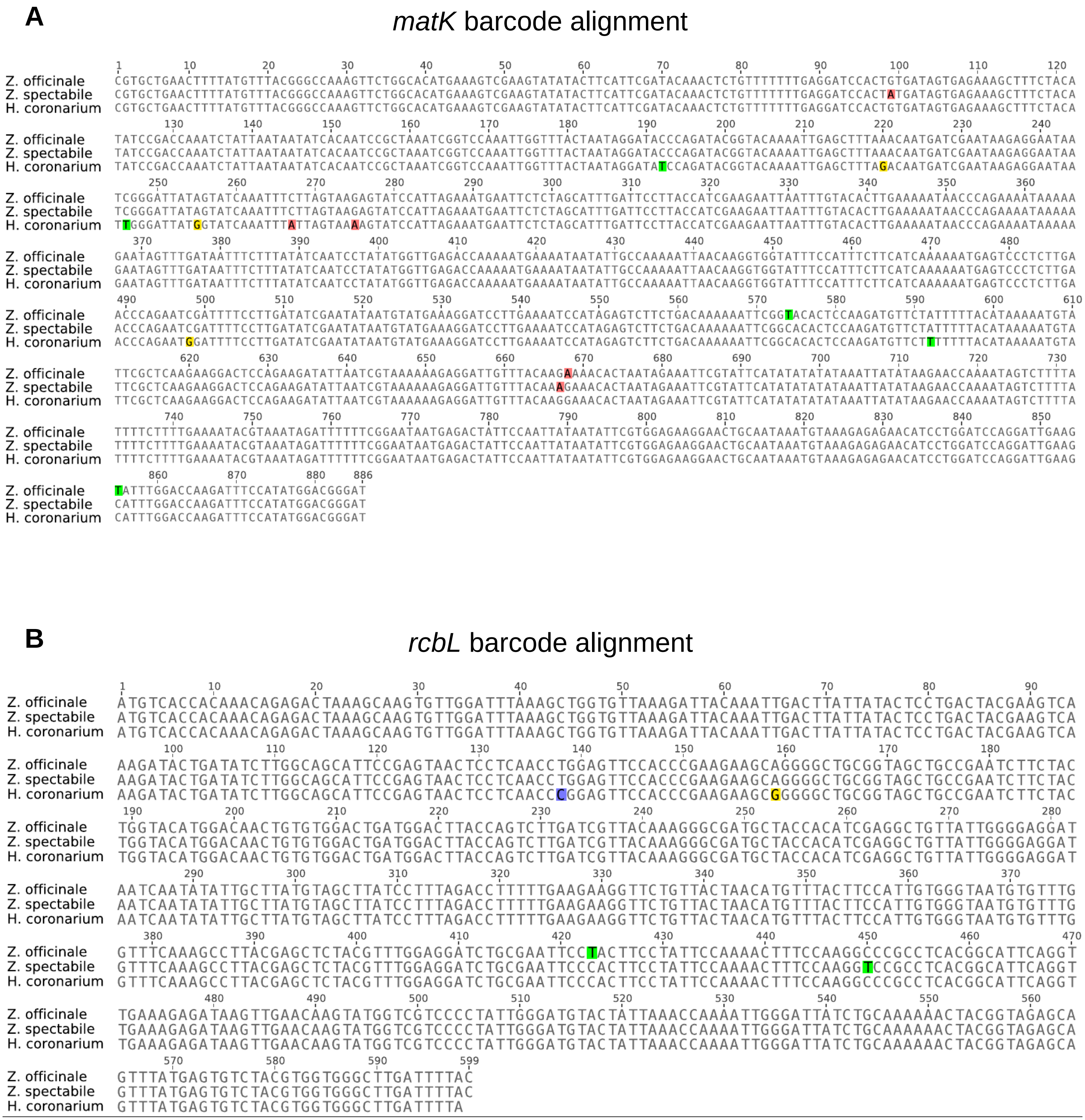
A
matK barcode alignment
Z. officinale
Z. spectabile
H. coronarium
B
rcbL barcode alignment
Z. officinale
Z. spectabile
H. coronarium
