# Supplementary material for: Whole Plastome Sequences from Five Ginger Species Facilitate Marker Development and Define Limits to Barcode Methodology
Source: PLoS One. 2014 Oct 21;9(10):e108581. doi: 10.1371/journal.pone.0108581 (PMC4204815; doi:10.1371/journal.pone.0108581)
Supplement: Figure S5 — Gel documenting amplification efficiency of JNV-A barcode sequence among Zingiberaceae accessions. Lanes are coded based on the matrix given below the gel. Plant accessions are as they appear in Table S2. (PDF) [file pone.0108581.s005.pdf]

JNV-A

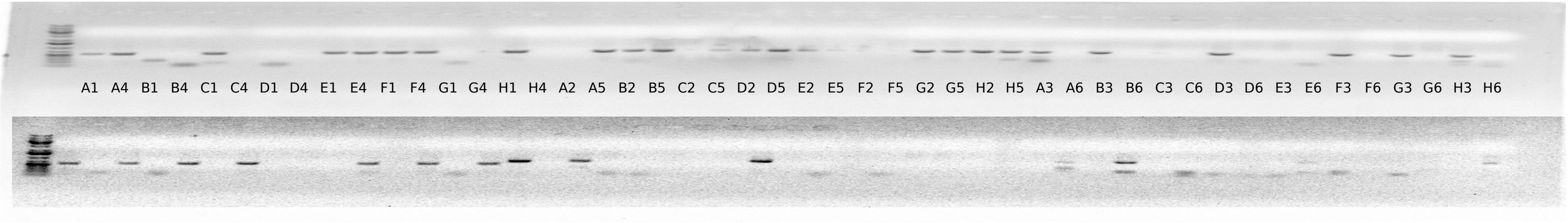

	1	2	3	4	5	6
A	ZMN05_Curcuma aromatica	ZSG03_Kaempferia pulchra	237.05_Hedychium coronarium	ZSA04_Alpinia nigra	G7_Zingiber officinale	**C6_Zus2**
B	ZMN19_Curcuma aromatica	ZSG01_Kaempferia galanga	ZMN09_Hedychium coronarium	ZSA03_Alpinia malaccensis	A8_Zingiber unknown	**D6_Zus9.1**
C	ZMZ10_Curcuma aromatica	ZSG02_Kaempferia angustifolia	ZSF04_Hedychium stenopetalum	ZMN01_Alpinia galanga	F4_Curcuma unknown	**E6_Zus1**
D	ZSD10_Curcuma zedoaria	ZSG04_Kaempferia rotunda	ZMZ22_Hedychium flavascens	ZMN03_Amomum species	E7_Emperor Trmeric	D7_Curcuma domestica
E	C. aromatica_Curcuma_Assa m	ZSB02_Amomum subulatum	ZSF01_Hedychium coronarium	ZMN10_Zingiber cassumunar	**E3_Zus1**	H7_Zingiber officinale 1_UGA
F	ZMZ05_Curcuma longa	ZSB01_Amomum species	ZMN12_Hedychium aurantiacum	ZMN04_Curcuma sp.	**H4_Zus8**	A8_Zingiber unknown _UGA
G	ZSD06_Curcuma longa	ZMN03_Amomum species	ZMN08_Alpinia species	ZSH01_Zingiber cassumunar	**B5_Zus10**	B8_Zingiber unknown _UGA
H	ZMZ04_Costus specious	ZSF05_Hedychium spicatum	ZMN01_Alpinia galanga	C4_Zingiber officinale_UGA	**B6_Zus3**	C8_Zus3
