# Supplementary material for: Whole Plastome Sequences from Five Ginger Species Facilitate Marker Development and Define Limits to Barcode Methodology
Source: PLoS One. 2014 Oct 21;9(10):e108581. doi: 10.1371/journal.pone.0108581 (PMC4204815; doi:10.1371/journal.pone.0108581)
Supplement: Table S1 — List of primers identified as optimal barcodes. Shown as ‘JNV barcodes’ in Figure 3. (DOC) [file pone.0108581.s006.doc]

**Table S1.** List of primers identified as optimal barcodes.

| **Barcode pair** | **Oligo Name** | **Sequence** |
| --- | --- | --- |
|  | MpanZing18F | ACGAAAGATTTATTCCCCCG |
|  | MpanZing27R | CCAAATTATGGTGTTGACGC |
|  | kress_e | GGTTCAAGTCCCTCTATCCC |
|  | kress_f | ATTTGAACTGGTGACACGAG |
|  | MpanZing25F | ATGAGGATGGGTCATTCGAG |
|  | MpanZing34R | GGTCATGTCATATAGGCCCG |
|  | Bzingiber14F | CATTGCTCTTGCTAATGCGA |
|  | Bzingiber14R | CCACCTTAACGACCGAAAAA |
|  | Bzingiber11F | TTGCACCTAAAAAGATTCTGTGA |
|  | Bzingiber12R | TCGAATCAACTCGTCTAGCTTTT |
|  | BpanZingib27F | TGAAATGCACCAATCCGTAA |
|  | BpanZingib27R | GCCGAACAATGCAAAAGAAT |
|  | BpanZingib21F | CGAGTCACACACTAAGCATAGCA |
|  | BpanZingib21R | GGAAGCATCGAAGAATTACAGG |
| B | BpanZingib10F | TCAAGTCCCTCTATCCCCAA |
| B | BpanZingib10R | CCCGGAAAGTCAAAGTAACG |
| A | BpanZingib2F | AATTGACCTCTACGGTCCCA |
| A | BpanZingib2R | GTGCTGGAACGTCCACTTTT |
