# Supplementary material for: Whole Plastome Sequences from Five Ginger Species Facilitate Marker Development and Define Limits to Barcode Methodology
Source: PLoS One. 2014 Oct 21;9(10):e108581. doi: 10.1371/journal.pone.0108581 (PMC4204815; doi:10.1371/journal.pone.0108581)
Supplement: Table S2 — Species list and results for barcode A and C amplification. (DOC) [file pone.0108581.s007.doc]

#### **Table S2: Species list and results for barcode A and C amplification.**

| S. No. | Accession Name | Species | Site of collection | PCR with barcode primers | |  |
| --- | --- | --- | --- | --- | --- | --- |
|  |  |  |  | A | C | |
| 1 | ZMN01 | *Alpinia galanga* | Assam, India | + | + | |
| 2 | ZSA03 | *Alpinia malaccensis* | Assam, India | + | + | |
| 3 | TARS17329 | *Alpinia purpurea* | Puerto Rico, US | + | + | |
| 4 | ZMN03 | *Amomum species* | Assam, India | + | + | |
| 5 | ZMN05 | *Curcuma aromatica* | Assam, India | + | - | |
| 6 | ZMN19 | *Curcuma aromatica* | Assam, India | + | - | |
| 7 | CA1 | *Curcuma aromatica* | Assam, India | - | + | |
| 8 | ZMZ05 | *Curcuma longa* | Assam, India | - | + | |
| 9 | ZMN04 | *Curcuma Sp.* | Assam, India | + | + | |
| 10 | CU1 | *Curcuma domestica (Emperor turmeric)* | Ohio, US | + | + | |
| 11 | TARS18166 | *Curcuma sp.* | Puerto Rico, US | + | + | |
| 12 | TARS1555 | *Etlengea sp.* | Puerto Rico, US | + | + | |
| 13 | ZMN09 | *Hedychium coronarium* | Assam, India | + | - | |
| 14 | ZMZ22_ | *Hedychium flavescens* | Assam, India | + | + | |
| 15 | ZSF04 | *Hedychium stenopetalum* | Assam, India | + | + | |
| 16 | ZSG02 | *Kaempferia augustifolia* | Assam, India | + | + | |
| 17 | ZSG01 | *Kaempferia galanga* | Assam, India | + | + | |
| 18 | ZSG03 | *Kaempferia pulchra* | Assam, India | + | + | |
| 19 | ZSH01 | *Zingiber cassumunar* | Assam, India | + | + | |
| 20 | ZO1 | *Curcuma sp.* | Ohio, US | + | + | |
| 21 | ZO2 | *Curcuma sp.* | Ohio, US | + | + | |
| 22 | PI194126 | *Zingiber officinale* | Puerto Rico, US | + | + | |
| 23 | TARS18100 | *Zingiber officinale* | Puerto Rico, US | + | + | |
| 24 | ZU1 | *Curcuma sp.* | Ohio, US | + | + | |
| 25 | ZU2 | *Curcuma sp.* | Ohio, US | - | + | |
| 26 | ZU3 | *Curcuma sp.* | Ohio, US | - | + | |
